# Poster Session I- A44 CHARACTERIZING THE NLRP6 INFLAMMASOME IN THE INTESTINAL EPITHELIUM

**DOI:** 10.1093/jcag/gwaf042.044

**Published:** 2026-02-13

**Authors:** L Li, N Winsor, D J Philpott, S Girardin

**Affiliations:** Laboratory Medicine and Pathobiology, University of Toronto, Toronto, ON, Canada; Laboratory Medicine and Pathobiology, University of Toronto, Toronto, ON, Canada; Laboratory Medicine and Pathobiology, University of Toronto, Toronto, ON, Canada; Laboratory Medicine and Pathobiology, University of Toronto, Toronto, ON, Canada

## Abstract

**Background:**

The intestinal epithelium serves as a critical barrier and interface between the external environment and the host’s internal milieu. It functions not only as a physical barrier but also as an active player in the innate immune system regulation. One member of the innate immune system are inflammasomes, which are multi-protein immune complexes found in the cytosol that assembles in response to PAMPs and DAMPs. One key member highly present in the intestinal epithelium is the NLRP6 inflammasome. In mice, they activate inflammatory caspases such as caspase 1, which in turn cleave and release inflammasome-derived cytokines, orchestrating the immune defense against pathogenic microbes and preserving the integrity of the gut barrier. Additionally, activated caspase-1 also cleaves the pore-forming protein GasderminD (GSDMD), leading to the release of GSDMD N terminus fragment (NTD). This fragment inserts itself into the plasma membrane, triggering pyroptosis, a form of cell death characterized by cellular lysis and release of inflammatory intracellular contents.

**Aims:**

Surprisingly, the mechanism of action and the direct ligand of the human NLRP6 inflammasome remains understudied, mainly due to a lack of model to study it in the human intestinal system, as NLRP6 is shown to be not present in human intestinal cell lines. In this study, we aim to develop a human based model to study the NLRP6 inflammasome using human intestinal organoids.

**Methods:**

We used human duodenal organoids, cultured in both 3D and 2D, to establish a model for investigating the NLRP6 inflammasome. Intestinal organoids are an *in vitro*, intestinal stem cell-derived culture system utilizing primary tissue samples. They are embedded in Matrigel, a gelatinous extracellular matrix rich in lamin, collagen, and growth factors essential for epithelial growth.

**Results:**

By seeding 3D cystic organoids into monolayers, we were able to induce NLRP6 expression successfully and reproducibly for the first time. Interestingly, the cell population also changes from a more stem cell rich pool toward differentiated enterocytes. In addition, combining this technique with lentiviral shRNA transduction, we were able to generate stable knockdowns of both NLRP6 and GSDMD in our organoids. Currently, we are using this knockdown system to study the NLRP6 system regulations.

**Conclusions:**

Developing a human-based organoid system to study the NLRP6 inflammasome pathway provides valuable insights and serves as a critical tool for understanding immune defense mechanisms against pathogenic microbes while maintaining gut barrier integrity. Currently, many auto-inflammatory and autoimmune diseases are linked to inflammasome dysregulation. Therefore, gaining a deeper understanding of the mechanism of NLRP6 could reveal critical intervention points in health and disease, given the vital role inflammasomes have in host defense.

**Funding Agencies:**

CIHRCGS-M

